# An Architecture for Managing Data Privacy in Healthcare with Blockchain

**DOI:** 10.3390/s22218292

**Published:** 2022-10-29

**Authors:** Anubis Graciela de Moraes Rossetto, Christofer Sega, Valderi Reis Quietinho Leithardt

**Affiliations:** 1 Federal Institute of Education, Science and Technology Sul-rio-Grandense, Passo Fundo 99064-440, Brazil; 2 COPELABS, Lusófona University of Humanities and Technologies, Campo Grande 376, 1749-024 Lisboa, Portugal; 3 VALORIZA, Research Center for Endogenous Resources Valorization, Instituto Politécnico de Portalegre, 7300-555 Portalegre, Portugal

**Keywords:** blockchain, cryptography, DApp, health data, privacy

## Abstract

With the fast development of blockchain technology in the latest years, its application in scenarios that require privacy, such as health area, have become encouraged and widely discussed. This paper presents an architecture to ensure the privacy of health-related data, which are stored and shared within a blockchain network in a decentralized manner, through the use of encryption with the RSA, ECC, and AES algorithms. Evaluation tests were performed to verify the impact of cryptography on the proposed architecture in terms of computational effort, memory usage, and execution time. The results demonstrate an impact mainly on the execution time and on the increase in the computational effort for sending data to the blockchain, which is justifiable considering the privacy and security provided with the architecture and encryption.

## 1. Introduction

Several advances related to blockchain technology have been recently consolidated, notably: the advent of blockchain 2.0, the blockchain network Ethereum (major programmable and public blockchain), the Hyperledger Fabric (private and permissioned blockchain), the improvement of smart contracts and the use of encryption on the data flowing through the blockchain, such as Elliptic Curve Cryptography (ECC) [1]. As a result, it has become possible to develop ways to ensure the privacy, integrity, and access control of the data within within a specific application in various scenarios and solutions as described [2,3].

According to Nakamoto [4], the data is publicly visible to everyone on the blockchain network. Consequently, it is important that this information is encrypted before being stored to ensure the confidentiality of the data and to keep the content private, helping to reduce the risk of the pseudonym being linked to the the real identity of the blockchain user, which is crucial to promote sharing based on the need-to-know. In addition, blockchain makes it possible to ensure that data cannot be deleted or tampered with [5].

To ensure privacy, in some cases it is necessary to use cryptography, which has several techniques and algorithms that can be used to implement the security it provides. Another aspect that must be considered is the identification of the type of cryptography that best satisfies the problem of ensuring the security of the data that will be stored in the blockchain. Among several examples of applications and scenarios, is the control of access to information such as medical diagnoses, test results, and other confidential, vital, and sensitive information to the patient should be under his responsibility, because in a centralized environment the patient has no control over the stored data, as only the institution that stores it. In this way, as pointed out in Ref. [6], the user does not know if his information may be made available to, for example, an insurance company that may not accept to perform certain coverage due to this improper disclosure of data.

Faced with the challenges of health data security, blockchain technology can significantly contribute in terms of user authentication, access control, and data privacy, as well as enabling the development of decentralized application (DApp). DApps run in a distributed way, that is, they do not need a central entity to coordinate the tasks [7]. Therefore, they prevent “data owners” from having to trust whoever centralizes the data and their ability to prevent private information leaks. The management of data privacy based on rules, parameters, and user definitions, and the computing environment, is based on the work developed in [8,9,10,11].

The motivation for this project came from papers that address the blockchain scenario, such as the papers of Zhang, Xue, and Li [5], Shi et al. [6], Dasgupta, Shrein, and Gupta [12] and Feng et al. [13]. These works point to healthcare-oriented systems as a potential to be researched, especially with the privacy layer. In this sense, this work presents a decentralized architecture, leaving the responsibility of the documents to the users, in order to guarantee the privacy of the data related to the health area that are stored inside a blockchain network and in the InterPlanetary File System (IPFS), through the use of cryptography with the Rivest-Shamir-Adleman (RSA), ECC, and Advanced Encryption Standard (AES) algorithms. Evaluation tests were also conducted to verify the impact of encryption on the architecture, considering the criteria of cost, memory usage, and runtime.

This paper is organized as follows: the Section 2 discusses the related works of applications for the health area. The Section 3 presents the concepts and characteristics of Blockchain and Smart Contracts. The Section 4 addresses the cryptographic algorithms used in the architecture. The Section 5 presents the tools needed to build the architecture. The Section 6 presents the proposed architecture along with its components. The Section 7 describes how the architecture was evaluated with the encryption algorithms and presents the results obtained. Finally, the Section 8 presents the final considerations and notes on the next steps of the research.

## 2. Related Works

The paper proposed by Aguiar et al. [14] presents the development of a blockchain framework using Hyperledger Fabric, a private and permissioned blockchain, along with anonymization methods used to preserve privacy during health data sharing. It is characterized as a different approach than the one proposed in this paper by the choice of the blockchain network and the use of anonymization methods such as K-Anonymity.

The work of Omar et al. [15] uses the MediBchain, which has some similarity because it seeks to leave the control of cloud data under the responsibility of the patient/user, making use of the ECC algorithm encryption to achieve pseudo-anonymity and privacy. The authors do not present the use of some other encryption algorithm, nor the use of IPFS to decrease the cost of storage in the blockchain, and also do not mention the possibility of allowing access to the files. An in-depth analysis of the cost and protocol of the developed system is presented.

In the work of Gan et al. [16] an access control is proposed, where patients will be supervisors of their medical data and institutions can access their data without authorization, but one can revoke access at any time. An incentive system is considered in the paper where patients who share their data receive a reward according to an evaluative criterion.

Liang et al. [17] propose a scheme to store personal data using a consortium blockchain, private and permissioned, and the Paillier homomorphic encryption mechanism, enhanced to ensure privacy and data protection. They also make use of off-chain storage with IPFS in the tests, which are aimed at evaluating the performance of the Paillier encryption mechanism.

Some applications of blockchain have even been used for artificial intelligence (AI) [18]. According to Dinh and Thai [19] there is a disruptive integration between AI and blockchain. Artificial intelligence has many applications, such as computer vision [20,21,22], time series forecasting [23,24,25,26,27], pattern classification [28,29], optimization [30], and recently some papers have been presented relating the topic to blockchain [31]. According to Atlam et al. [32], besides the application of blockchain in AI there is room for its application in the internet of things.

In Ref. [33], a data protection scheme with attribute-based encryption (CEC-ABE) combined with a blockchain is proposed to protect electronic health records in edge cloud environments. In the tests, the proposed algorithm (CEC-ABE) is compared with other algorithms, for example CP-ABE, to evaluate the performance in the proposed architecture by checking the computational cost of each stage (global initialization, key generation, plaintext encryption, outsourced decryption, and final decryption).

Shahnaz, Qamar, and Khalid [34] presented a framework for the health sector to perform secure storage of electronic records in the blockchain with access rules, using IPFS to store the files. However, the work does not focus on privacy and encryption of data to ensure security, since the security of the presented system depends on the very security provided by the technologies used, unlike this work, which aims to ensure privacy with the addition of one more encryption scheme. Tests are also performed simulating a scenario with several users using different functions of the framework, where three criteria are evaluated: execution time, transfer rate, and latency.

Table 1 presents a comparison between the works cited above and our work regarding some characteristics. Importantly, none of the works employ an evaluation of the performance of the ECC and RSA cryptography algorithms in the context of a blockchain architecture. Among the works that employ the use of cryptography along with the characteristic of being a DApp to ensure data privacy, only this work and [17] make use of IPFS as an off-chain mechanism, however the latter is not a proposal for the health area.

## 3. Blockchain and Smart Contracts

Blockchain was originally introduced, or received more recognition, when Nakamoto in his work proposed a financial system using blockchain to record all transfers of the digital currency Bitcoin securely and reliably [13]. This technology is a decentralized, distributed, and immutable ledger, consisting of a collection of records that are cryptographically linked. Such a collection is better known as a blockchain that stores transactions or events [35]. This ledger is shared with all participating members (nodes) of the blockchain network.

Transactions that are performed between members of a blockchain must be approved by the mining nodes before they can be confirmed and added to the blockchain network. Thus, to start the mining process, the transaction is transmitted to all nodes in the network and the nodes that are miners will organize the transactions into a block, verify the transactions in the block, and transmit the block and its verification using a consensus protocol, for example Proof of Work (POW), to get the network’s approval [5]. Once the other nodes have verified that all the transactions contained in the block are valid, the block can be added to the blockchain via a cryptographic hash function that connects the blocks in the framework, where the hash of the *n* block is linked to the hash of the *n + 1* block [36].

Among the features of blockchain, the most important ones according to Hewa, Ylianttila, and Liyanage [35] are:

- Decentralization: grants authority to network members, ensuring redundancy in contrast to centralized systems operated by a trusted third party. Decentralization reduces the risk of failures and ultimately improves service reliability with guaranteed availability;
- Immutability: the transaction records in the ledger, distributed among the nodes, are permanent and unchangeable. Immutability is a feature that differs from centralized database systems. The records are resistant to computational tampering with the existence of cryptographic links;
- Cryptographic Link: the cryptographic link between each record is sorted in chronological order, building an integrity chain across the blockchain. The digital signature verifies the integrity of each record using hashing techniques and asymmetric key cryptography. Violating the integrity of the blockchain record or transaction ultimately renders the record and the block invalid.

Blockchain security is part of the advances in cryptography and blockchain design and implementation (Bitcoin, Ethereum, etc.). Blockchains have been made proposals over time to improve the efficiency of the cryptographic blockchain, for example, incorporating Merkle trees and putting multiple documents in a block [5]. The blockchain was built to guarantee several features regarding security, such as consistency, tamper resistance, pseudo-anonymity, and resistance to double-spend and Distributed Denial of Service (DDoS) attacks. However, even with the current level of security that blockchain can provide, in some scenarios additional security and privacy properties are still lacking [5].

Smart contracts can be thought of as a program that is executed when predetermined conditions are met (self-executing) and is deployed on the blockchain, and can be used in financial services, healthcare, and government. It is capable of supporting complex programmable functions and mechanisms to automate agreements and other types of [6] flows. This type of contract, which can be used on the blockchain, allows the parties to make use of it to create trusted virtual third parties who have behaved according to the rules agreed upon between them, thus allowing the creation of complex protocols with a very low risk of noncompliance [37].

In the Ethereum blockchain network, smart contracts are formally developed into high-level code via Solidity (a contract-oriented programming language similar to JavaScript) and are compiled to be executed by the Ethereum Virtual Machine (EVM). In the concept of Solidity, smart contracts are a set of code, data, functions, and states, which are at a specific address on the Ethereum blockchain network [38].

For an account to interact with a contract or for interactions to occur between contracts, the name and arguments of the function must be known. This gives rise to the Application Binary Interface (ABI), which is a list of the functions and arguments of the contract organized in a JavaScript Object Notation (JSON) format, as soon as it is compiled. The ABI is then used to hash the function definition and then create the EVM bytecode needed to call the function [39].

## 4. Encryption Algorithms

Cryptographic algorithms can be split into two types, symmetric key or private key, which can be further divided into algorithms that operate on a single bit or groups of bits, and asymmetric key or public key algorithms. Public key cryptography was created in 1976 when W. Diffie and M. Hellman, proposed this new idea, which was followed by R. L. Rivest, A. Shamir, and L. Adleman, the creators of the RSA algorithm [40].

According to Singh, Khan, and Singh [41], these are algorithms that rely on the use of a public key and a private key. The public key will be freely distributed without compromising the private key in any way, which must be kept secret. The public key is used to encrypt plaintext messages and verify signatures, while the private key is used to sign messages and decrypt the ciphertexts to obtain the plaintext messages. Elliptic curve-based encryption, which is a special type of public key, was first proposed by Miller and Koblitz in the late 1980s, and was based on previously existing public key algorithms and applications [40].

The symmetric key algorithms will have a key (private) that is equal for both parties that are exchanging information and must remain secret. Because it will be used to encrypt and decrypt the information, it is a simple method that facilitates encryption. However, the problem with this method lies in the sharing of the key between the parties, because if someone manages to intercept this exchange and gain access to the key, that person will have access to encrypt and decrypt the information [42]. Cryptography provides a mechanism to secure data in today’s information systems. This work used the AES algorithm in conjunction with RSA and ECC, to create a hybrid cryptosystem with the goal of increasing the complexity and strength of cryptography. The RSA, ECC, and AES algorithms are discussed below.

### 4.1. RSA

The asymmetric key RSA encryption algorithm has become the standard for public key cryptography and is widely used. Its security lies in the integer factoring problem and its decryption process is not as efficient as its encryption process. For better and stronger data security, RSA ends up needing larger key sizes, which implies more overhead on systems. So for systems with memory constraints, RSA becomes a second option [43].

### 4.2. ECC

The security of the asymmetric key ECC encryption algorithm lies in the use of the mathematical properties of the elliptic curve to perform the calculation of the cryptographic keys (discrete logarithm problem on elliptic curves). It is an adequate and promising system for devices that have memory constraints (Smartphones and Smartcards), besides being used in blockchains, such as Bitcoin, which has the highest market value today. ECC can maintain security levels equivalent to RSA and requires comparatively fewer parameters for encryption and decryption than RSA [43].

### 4.3. AES

The AES symmetric key encryption algorithm was developed in 1998 by Joan Daemen and Vincent Rijmen. It allows a fixed data block size of 128 bits and supports key sizes of 128, 192, and 256 bits, and any combination of data [44]. According to Oliveira [42], AES is one of the most popular symmetric key algorithms, being adopted as a standard by the United States government, and is considered as the replacement for the Data Encryption Standard (DES) due to its speed, easy execution, and low memory requirements.

Being the most widely used symmetric key block cipher within computer security, mainly by its standardization by NIST and also by all published cryptoanalysis on this algorithm, it is able to resist several types of attacks. Therefore, it becomes an ideal choice for encrypting a larger volume of data, due to its performance, and can be combined with the security of an asymmetric key algorithm [45].

## 5. Tools

For the development of the solution, the tools and technologies discussed in the next subsections were used.

### 5.1. Truffle

Truffle is a development environment, test framework, and asset pipeline for blockchains using the Ethereum Virtual Machine (EVM) [46]. According to the website [47], the features that the developer gets to enjoy when using Truffle are:

- Integrated binary smart contract compilation, binding, deployment, and management;
- Automated contract testing;
- Programmable and extensible deployment and migration framework;
- Network management for deployment in public and private networks;
- Package management with EthPM & NPM, using the ERC190 standard;
- Interactive console for direct contract communication;
- Configurable build pipeline;
- External script executor that runs scripts in a Truffle environment.

### 5.2. Ganache

Ganache allows its user to have a personal blockchain for DApps development, enabling the developer to design, deploy, and test their DApps in a secure and deterministic environment. It is possible to develop for the Ethereum and Corda network. Furthermore, through Ganache you can test how your DApp affects the blockchain and examine details such as your accounts, balances, smart contract creations, and gas costs [48].

### 5.3. MetaMask

MetaMask is an encrypted (digital) wallet and gateway to blockchain applications that enables users to manage their accounts, keys, and tokens in a variety of ways, including hardware wallets, and isolates the user from the context of the website. It is available as a browser extension and as a mobile app [49].

For developers, it is possible to interact with Ethereum’s (globally available) API that identifies users of Web3 compatible browsers. Whenever a request for a transaction signature happens, MetaMask will prompt the user for a confirmation of that transaction as well as indicate the cost of it. MetaMask is already configured with some connections to the Ethereum blockchain network and to various test networks via the Infura API. In addition, MetaMask is currently compatible with any other blockchain (public and private) that exposes a JSON RPC (Remote Procedure Calling) API compatible with Ethereum [49].

### 5.4. React

React is a JavaScript library used for building user interfaces. This library is declarative, which makes your code more predictable and easier to debug. It is component-based, which makes it easy to pass various types of data throughout your application and still maintain a state outside of the Document Object Model (DOM). React components implement a ”render()” method that will receive input data and return what should be displayed. In addition, a component can maintain internal state data. React makes it easy to interface with other libraries and frameworks [50].

### 5.5. Node.js

Node.js is as an asynchronous, event-driven JavaScript runtime designed to create scalable web applications. Node.js users do not need to worry about process locking, because almost no functions in Node.js directly execute input and output, so the process is never locked up, except when using synchronous methods from the Node.js standard library to execute input and output [51].

Influenced by systems such as Ruby’s Event Machine and Python’s Twisted, Node.js introduces an event loop as a runtime construct. As there is no call that will initiate the event loop, it simply enters the event loop after executing the input script and exits the event loop when there are no more callbacks to execute [52].

Even if it was designed without threads, it is possible to make use of several cores of an environment, through the child processes that can be generated using the childprocess.fork() API. Using the cluster module, which makes it possible to share sockets between processes and thus load-balance their cores [51]. Therefore, Node.js is a common choice for developing scalable applications, and in the case of DApps development this platform is also much preferred in conjunction with React, thus developing the front-end and back-end in JavaScript.

### 5.6. WEB3

WEB 3.0 focuses on decentralization, unlike WEB 1.0 and WEB 2.0, and also brings some additional features such as being verifiable, self-governed, permissionless, and distributed. Web3 applications (DApps) run on decentralized networks, blockchains or even a combination of the two forming, for example, a cryptoeconomic protocol, as cryptocurrency plays a big role in many of these protocols, providing a financial incentive (tokens) for nodes that want to participate in the creation, governance, contribution, or enhancement of a project [53].

The projects that are developed on top of this Web3 system end up offering a variety of services such as computing, storage, and hosting among other services that were mainly provided by cloud providers. The users that consume these Web3 services pay to use the protocol, but in this case the money goes directly to the network participants, eliminating unnecessary intermediaries [54].

In developing the DApp of this work, the Web3 library was used, which is employed to connect to the Ethereum network from an application. The ABI is given to the Web3 library, which it uses to give programmatic access to the deployed contract, which in this case is the Ethereum network. An object of the Web3 class must be instantiated to make use of its functions. Each instance of the Web3 library can connect to a different Ethereum network. The Web3 instance requires a communication layer known as a provider, which acts as a medium between the Web3 library and the Ethereum network, and each provider has a set of methods for sending or receiving a request from the Ethereum network [55].

### 5.7. Infura API

Infura’s API is powered by a microservices-oriented architecture that scales dynamically by providing instant access over Hypertext Transfer Protocol Secure (HTTPS) and WebSockets to the Ethereum network, providing an infrastructure for DApps easily and quickly. Through Infura, developers can connect to Ethereum and the IPFS via HTTPS and WebSocket with satisfactory response times and availability [56]. In addition, the platform provides a dashboard that shows the application performance and API usage, detailing specific method requests, usage time, and other features that can help the developer while building their application [57].

Some features available through Infura’s API according to the platform’s website [58]:

- Supports the mainnet and testnets via client-supported JSON-RPC, HTTPS and Windows Sharepoint Services (WSS);
- Works with the latest network updates with a minimum 99.9% uptime guarantee;
- Connects your application with one line of code without synchronization and complicated configuration;
- Allows you to configure, monitor, and analyze your applications with the Infura control panel;
- Access to Ethereum archive node data available as an add-on;
- 24/7 access to Infura’s expert support teams and experienced developer community.

## 6. Architecture

To accomplish health data privacy protection and verify the performance of encryption techniques, an application architecture was designed that allows the user to access and communicate with the Ethereum blockchain and IPFS, thereby obtaining their health-related data, which has been securely stored through encryption. The user can access the application by accessing MetaMask by adding a digital wallet. Users of the application can search for their stored documents and share documents. The persistence of the bulk data is done via IPFS.

The functional and non-functional requirements for the proposed solution were defined. As functional requirements, there are:

- Login: the application will only be available after the user login through MetaMask;
- Enter encryption keys: every time the user logs in to the application, his keys (public and private) will be generated. The application has a space for the user to enter his keys. The first time he accesses the application he can save his keys as he prefers, and the next time he logs in he can enter his keys;
- Store information: the user can send information to be stored in the IPFS, securely through AES encryption, which generates a hash to access the content on the network. This hash and the private key of AES, randomly generated, will be stored encrypted in the Ethereum blockchain, through one of the asymmetric key algorithms (ECC or RSA), to ensure privacy and access control to data;
- Confirm or reject transactions: the user, after sending a request to store information or a permission on the respective contracts, can accept or reject the transaction through MetaMask;
- Fetch information: the user can fetch the information they have stored in the IPFS through the hash stored in the blockchain, according to their logged in user;
- Grant permission: user A can grant access permission to a file that he has stored in the IPFS to user B, through some information such as the hash stored in the blockchain, the specific time that the permission will be valid, the address of user B, and the public key of user B;
- View files with permission: a user that has been granted permission by another user to a file, can have access to view that file within the time that was set by the user that granted the permission.

Regarding the non-functional requirements, the following were listed:

- DApp: the application will have the characteristics of a decentralized application;
- Application storage: the application does not store any data permanently and centrally, only temporarily;
- Data security: files sent to IPFS will be encrypted by the AES algorithm. The hash, which identifies the location of the data, and the AES private key (used to encrypt the file) will be encrypted using RSA or ECC before being stored in the blockchain, thus allowing the user to securely reaccess their data. The private key shared in the permission will be encrypted by AES.

**Figure 1:**
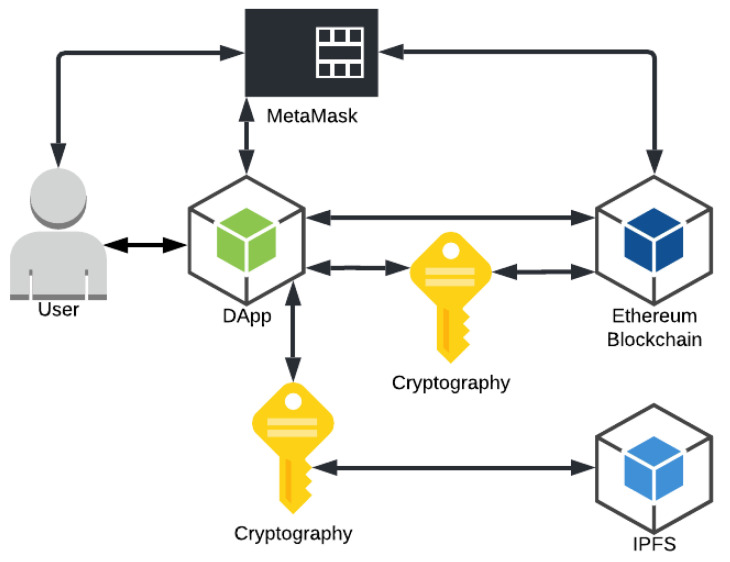
Architecture schema.

Figure 1 shows the solution architecture with the information exchange flows between the six components. The following describes the components of the architecture:

- User: will access the application, send and receive data (information, files and permissions), as well as confirm transactions and pay the persistence fee for this data in the blockchain;
- DApp: responsible for communicating with the digital wallet interface (MetaMask), with the blockchain (Ethereum) sending and receiving the data, with the decentralized bank (IPFS) sending the files and handling the return hash, and with the user receiving and showing the requested data, and, finally, perform the encryption/decryption of the hash, the AES private key, the file, and the shared AES private key when granting permission;
- Digital Wallet Interface (MetaMask): responsible for managing the transactions performed in the DApp, requesting user confirmation and debiting the fee for persisting the data in the blockchain;
- Blockchain (Ethereum): responsible for keeping the smart contracts that govern how the pertinent information of the file and the permission will be stored, such as, for example, the hash that is used to access the file in the IPFS, the address of the user who performed a transaction and the address of the user to which permission is being granted, thus performing the link of the respective user to certain information within the smart contracts;
- Decentralized file system (IPFS): responsible for receiving the encrypted files from DApp, storing them and returning a respective unique hash that indicates where this certain information is in the IPFS network;
- Encryption: this is the key component needed to ensure the security and privacy of the data stored in the proposed architecture, because through the RSA or ECC encryption algorithms the application encrypts the hash and the private key before sending this information to the blockchain. With AES the file and the shared private key are encrypted when granting permission. In this way, only the user who has access to the private key to decrypt this hash and the AES private key will be able to access the stored information. This process ensures that the information on the blockchain and IPFS is secure.

**Figure 2:**
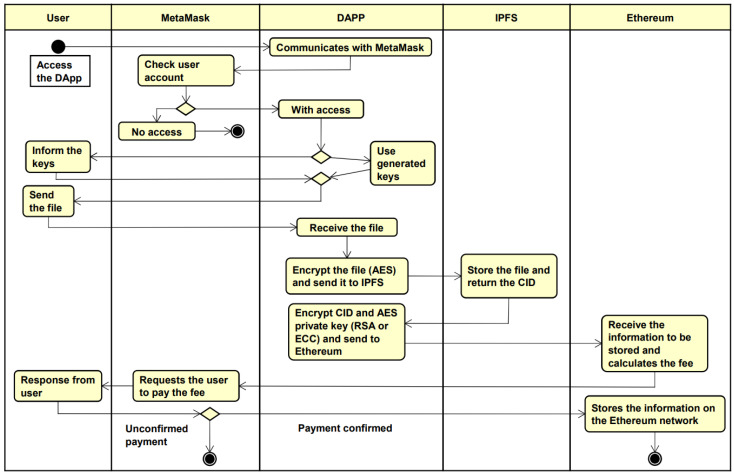
Architecture activity diagram.

The activity diagram in Figure 2 seeks to demonstrate the interaction that occurs between the components for sending a file.

Initially, the user opens the DApp and is required to login through MetaMask to access the DApp functionalities. After the login, DApp will use the cryptographic keys entered by the user or the cryptographic keys generated by DApp itself to encrypt the information. Next, the user selects the file to be sent, the DApp encrypts the file with the AES algorithm and sends it to be stored in the IPFS, which returns the Content Identifier (CID), a hash that identifies where the user’s file is in the IPFS network.

In this way, DApp encrypts the AES private key, used to encrypt the user’s file, and the IPFS CID with one of the algorithms (RSA or ECC). So with the sensitive information protected, it can be stored in the Ethereum blockchain, which prompts the user, via MetaMask, to pay the processing fee for the data to be stored. The user can then either reject and cancel the process or make the payment and have the data stored.

The operation of permissions is similar to the operation of sending a file, however, it is not necessary to send another file to IPFS, because it is done by sharing the necessary information for user B to be able to access the file of user A, such as the CID and the private key of AES. Thus, user A can send a permission by providing some information, such as user B’s public key, user B’s digital wallet address, and the permission expiration time set by user A.

### 6.1. DApp Implementation

This section presents details regarding the implementation of the DApp. The front-end and back-end of the DApp were developed with JavaScript, HTML5, React and Node.js, and the smart contract part in Solidity. The code (Code available at: https://github.com/SegaBR/DApp, accessed on 12 July 2022) for building the DApp was adapted from reviews made of the documentations of the technologies described, some decentralized open source projects developed by [59] and also searches through the cryptographic library documentation. More details about the architecture implementation are available at [60].

#### 6.1.1. Communication with Blockchain

**Figure A1:**
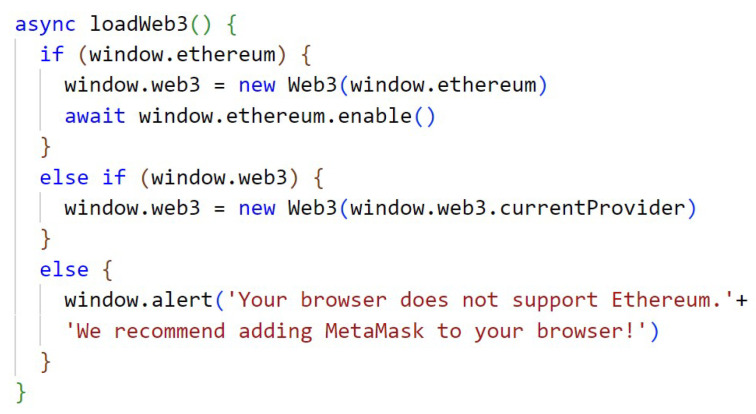
Communication with MetaMask.

Initially it is necessary to establish communication with a gateway to the Ethereum blockchain network, in this case MetaMask, to then establish communication with the blockchain. The Figure A1, in Appendix A, shows the code snippet responsible for this communication, and it is necessary to import the Web3 library into the project.

**Figure A2:**
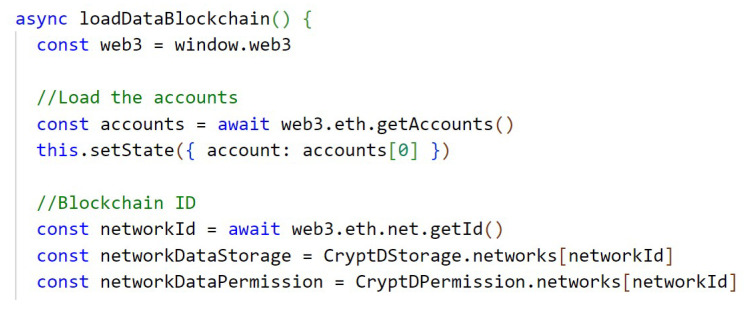
Loading account, fetching network ID, and contract data.

After establishing the previous communication, the loading of the blockchain data is performed. In Appendix A, Figure A2, it searches for the connected accounts, the blockchain network identifier, and the contracts deployed in the network, which in this case are the CryptDStorage, which is the contract responsible for the storage logic, and the CryptDPermission, which is the contract responsible for the permissions logic.

**Figure A3:**
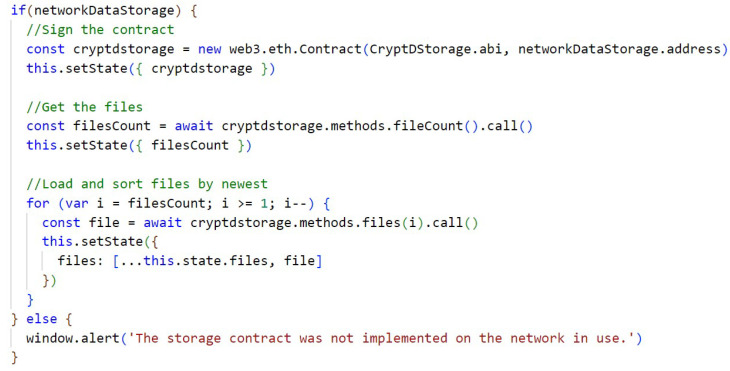
Loading contract data.

In Appendix A, Figure A3, we get the smart contract as if it were a JavaScript object so that we can store it in the state of the application and perform the search for the stored data associated with the smart contract; in the case of this code, the search is performed for the data of the CryptDStorage contract that is responsible for the file storage logic.

**Figure A4:**
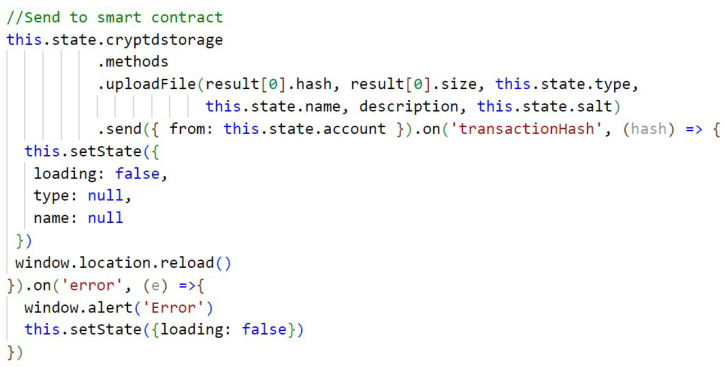
Sending data to the contract.

In Figure A4 (Appendix A) a call is made to the CryptDStorage smart contract, which sends the storage of a file to the blockchain, calling the “uploadFile” function of the smart contract and sending the necessary information that the function will use. The “send” method that is called will indicate which user is making the request and will pay the fee required for the Ethereum network to persist the data. The transaction is finalized when the transaction hash is generated.

#### 6.1.2. Communication with IPFS

**Figure A5:**
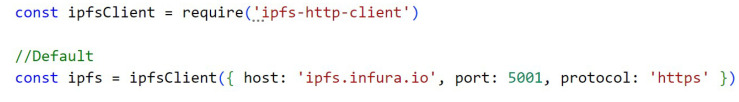
IPFS initialization.

To establish communication with IPFS, first an initialization is performed, as can be seen in Appendix A, Figure A5, where the data is informed to establish the communication that will be performed through Infura’s API.

**Figure A6:**
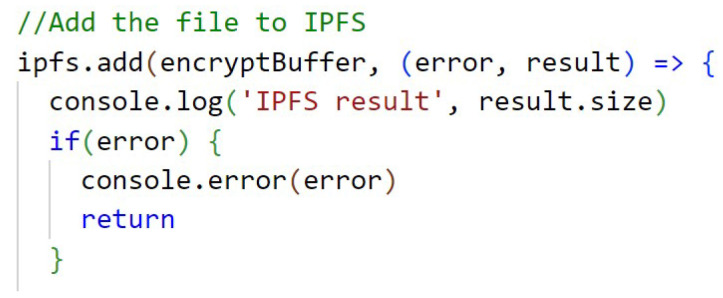
File upload to IPFS.

Although the code of Figure A6 (Appendix A) is sent a file to be stored on the IPFS, the variable result returns the hash, which is the CID that is used to fetch this content from within the IPFS.

**Figure A7:**
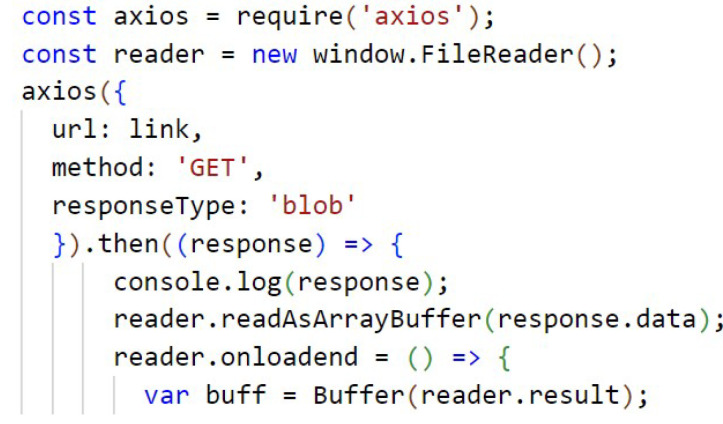
Search file in IPFS.

The code from Figure A7 (Appendix A) is responsible for the search of a file stored in the IPFS, through the link formed by the hash. The search is done using Axios, which is a HTTP client based on promises for node.js, which uses XMLHttpRequests. This retrieves the file and transforms it into a buffer so that it can be decrypted and made available for the user to access.

#### 6.1.3. Cryptography

Regarding the cryptography implemented in the application, it comprises three algorithms: RSA, ECC, and AES.

##### RSA

**Figure A8:**
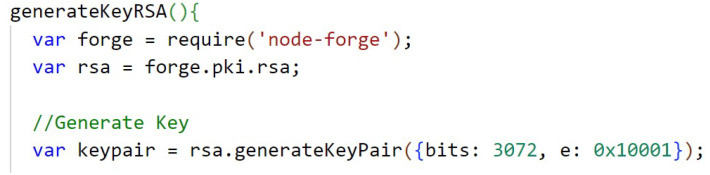
Generate RSA Keys.

**Figure A9:**
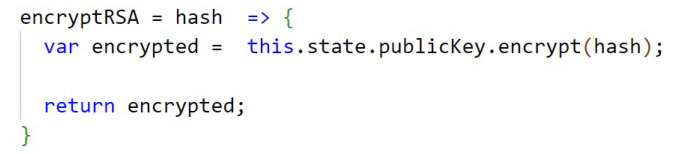
Encrypt RSA.

**Figure A10:**
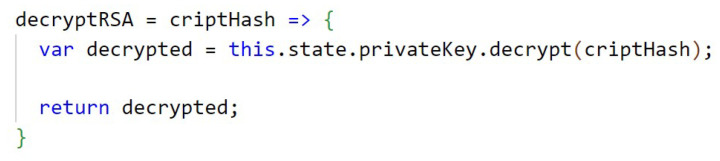
Decrypt RSA.

The Figure A8, in Appendix B, presents how the 3072 bit keys of the RSA (public and private) algorithm are generated, using the library node-forge. The code from Figure A9, in Appendix B, shows the function that performs the encryption of the data with RSA. In Appendix B, Figure A10, the function that performs the RSA decryption is shown. The keys are stored temporarily in the application’s state.

##### ECC

**Figure A11:**
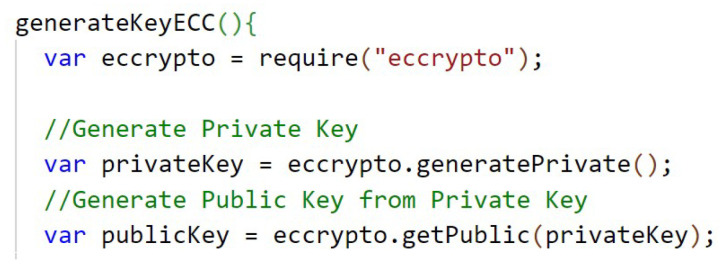
Generate ECC keys.

The code presented in Appendix C, Figure A11, shows the generation of the 256-bit keys of the ECC algorithm, first the private key and then the public key, using the library ecccrypto, the Elliptic Curve Integrated Encryption Scheme (ECIES) implementation and the elliptic curve secp256k12.

**Figure A12:**
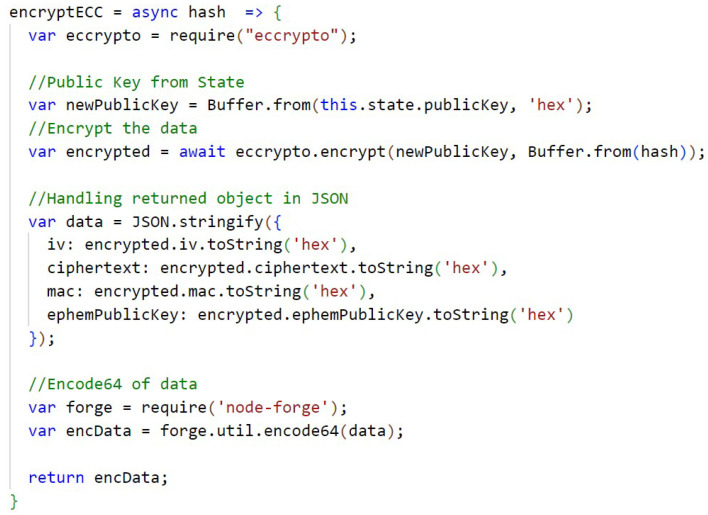
Encrypt ECC.

**Figure A13:**
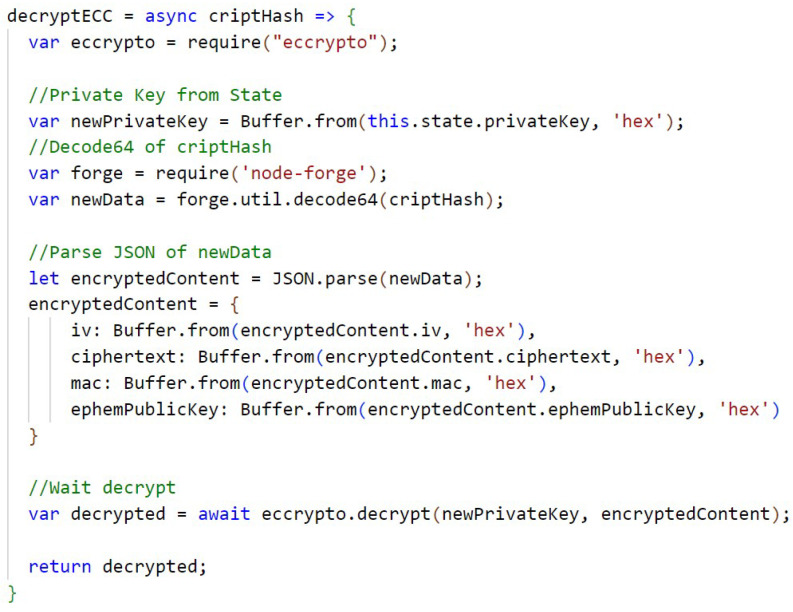
Decrypt ECC.

In Figure A12 (Appendix C) is the function that performs the encryption with ECC and the Figure A13, in Appendix C, has the function responsible for decrypting with ECC. The keys are temporarily stored in the state of the application. Because of the way ECC is implemented in the library, it is necessary to perform special handling on the returned object, in this case in JSON format, and perform an encode64 of the result to compress the information in the encryption function, and a decode64 of the result to decompress the information in the decryption function.

##### AES

**Figure A14:**
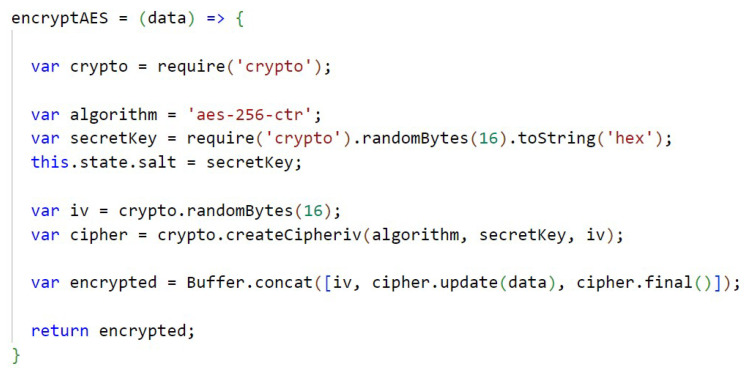
Encrypt AES.

**Figure A15:**
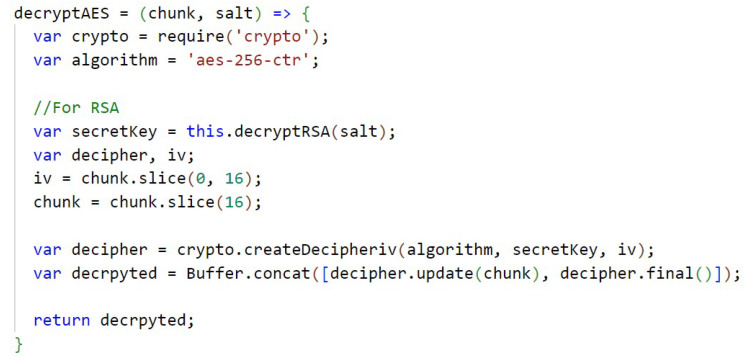
Decrypt AES.

In Figure A14 (Appendix D) the AES encryption with a key size of 256 bits and through the crypto library is shown. The key used to encrypt the files is also randomly generated using the crypto library. In Appendix D, Figure A15, the AES decryption is performed and, according to the algorithm used, decryption of the secret key is performed.

## 7. Architecture Evaluation

An evaluation of the architecture was conducted to verify the impact of using encryption with the two algorithms: RSA and ECC. The goal is to analyze the performance of the whole proposed architecture together with AES, considering the implication of encryption: (a) of the hash that IPFS returns to the application, (b) of the private key of AES used in the encryption of the file by the AES algorithm, and also (c) of the private key of AES and the CID that is shared in the permission. This encrypted information is stored in the blockchain.

The algorithms were evaluated regarding the criteria of execution time, memory usage, and the influence on the cost increase of the rate of sending information to the blockchain network. The evaluation does not consider the time to send the files to the IPFS, because many variables, such as internet connection, bandwidth, network traffic, among other unstable factors, can affect the process and make the results inaccurate.

ECC is an algorithm that is widely used in blockchain systems, such as the Bitcoin and Ethereum network. RSA is an algorithm that has become a mainstay and has been widely used for secure data transmission in various systems [61]. The evaluation of these two asymmetric algorithms, which are widely used in today’s systems, thus has the scope to show how they will perform within the proposed architecture, mainly due to the required key size characteristic.

According to recommendations by the National Institute of Standards and Technology (NIST), the 3072-bit key size for RSA matches the 256-bit key size for ECC in security, providing a 128-bit level of security, which is optimal for systems beyond the year 2030 [62]. Therefore, the paper adopts these key sizes. For ECC it makes use of the Elliptic Curve Integrated Encryption Scheme (ECIES) (ECIES: a hybrid encryption scheme that combines ECC-based asymmetric and symmetric encryption to provide data encryption via the corresponding ECC private key and public key) and the secp256k1 curve.

The same equivalent test parameters were used for each algorithm, and three different file sizes were sent: 200 KB (representing a simple examination report file), 2 MB (representing a more complex report or simple image file), and 30 MB (representing a more complex image file). The criteria evaluated for each algorithm, as well as for the unencrypted approach, are:

- Execution time: the measured time from the beginning of sending a file or permission until the end of the process, through the Truffle framework, which allows testing the sending of information to the blockchain and the Performance ( Performance: an interface that supports client-side latency measurements in applications, also present in Node.js, allowing the use of the “performance.now()” method that represents the time with floating point numbers with precision of up to microseconds.), which provides performance information related to time;
- Memory usage: through native features of Node.js itself, which provides information about memory consumption along with the Truffle framework;
- Impact of cryptography on the rate cost: we used the information provided by Ganache and MetaMask, which show the “gas” used to perform the transaction.

The environment for performing the tests consists of Ganache, creating a simulation of the Ethereum blockchain locally without connection to the main or testing network, and Truffle for running the local tests, thus obtaining the memory information, execution time, and approximate cost. The configurations of the machine used for processing the tests are: Intel(R) Core(TM) i5-8400 CPU—2.80 GHz, 8 GB RAM, and Windows 10 Pro operating system.

Before running the tests, simulating the user’s interaction with the application, the contracts were migrated to the Ganache test blockchain. The tests are based on sending a file or sending a file permission, where the information is treated and encrypted, and the information sent to the blockchain is confirmed and validated.

**Figure 3:**
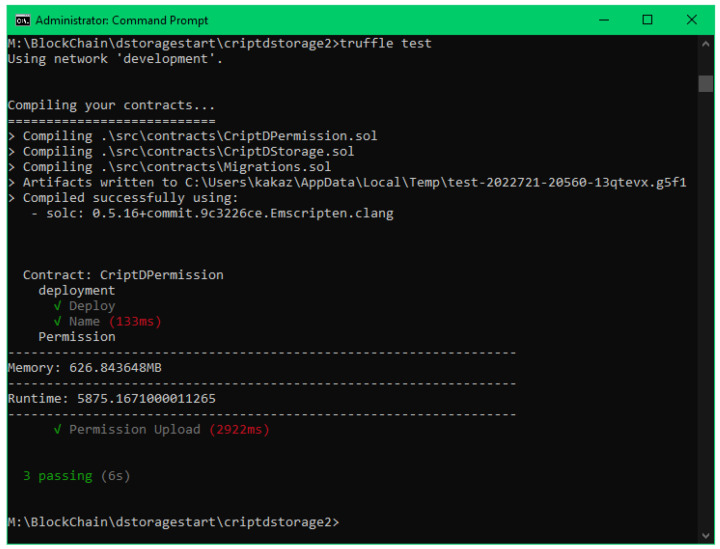
Result of running a test.

An example of a test run of sending a file permission using RSA encryption can be seen in Figure 3. The pseudocode for the test file can be seen in Algorithm 1.
**Algorithm 1:** Test file algorithm of submitting a file.1:Initialization of variables2:AES encrypt of the file3:RSA/ECC encrypt of the Hash and Secret Key4:Call and wait the event of the upload file “criptdstorage.uploadFile(args)”5:Check the information sent6:Show memory and runtime results

### Evaluation Results

**Figure 4:**
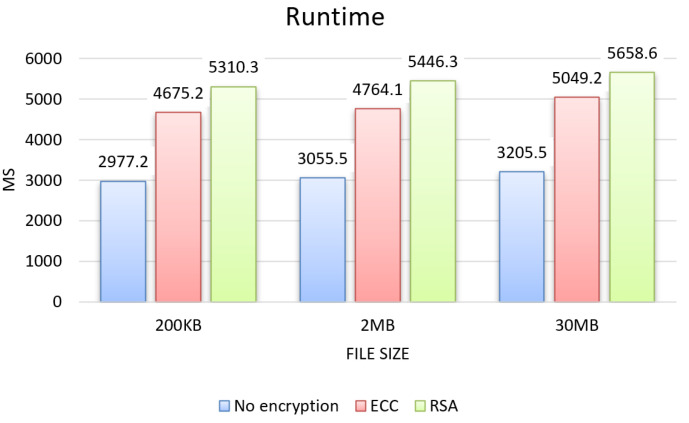
Runtime.

**Figure 5:**
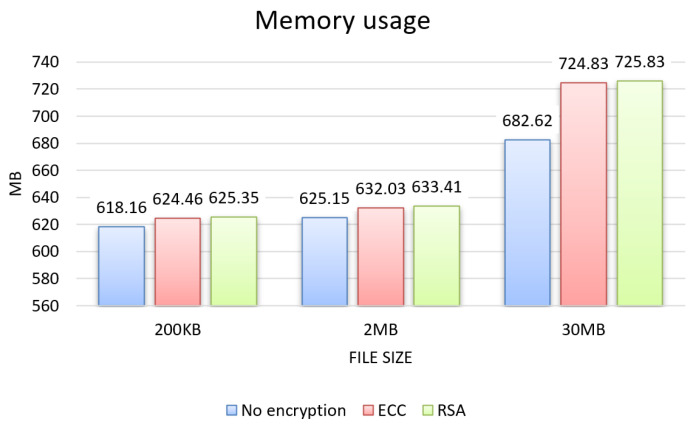
Memory usage.

**Figure 6:**
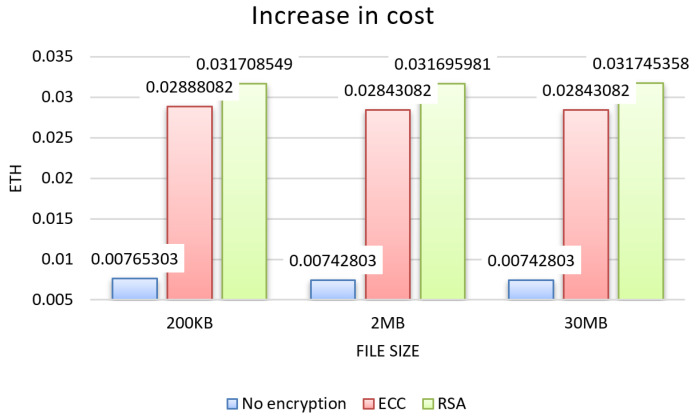
Increase in cost.

**Figure 7:**
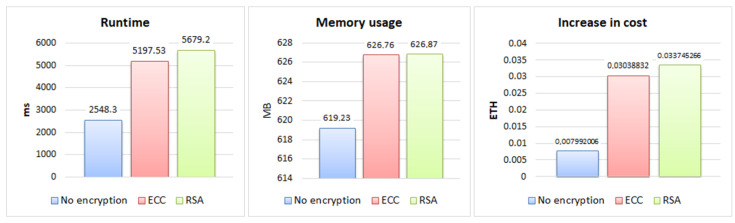
Results of submitting a file permission.

From the tests, the results for the three evaluation criteria were obtained, which can be seen in the graphs in Figure 4, Figure 5 and Figure 6 for each size of file sent 200 KB, 2 MB, and 30 MB, and in the graphs in Figure 7, showing the results about sending permission.

Regarding the runtime results, presented in the graph in Figure 4, both RSA and ECC showed an increase over the unencrypted approach for all three file sizes, RSA with an average increase of 75%, and ECC with 55%, with the largest increase for RSA with an average difference of 20% compared to ECC.

The evaluation regarding memory usage, presented in the graph in Figure 5, highlights a similar increase for the 200 KB and 2 MB file sizes compared to the unencrypted approach, with an average increase of 1%, representing 7 MB. The memory increase was most significant for the 30 MB file size, with 6%, representing 42 MB on average.

In the results regarding cost, verified in the graph in Figure 6, the increase was similar for the 200 KB, 2 MB, and 30 MB file sizes. Overall, the cost had an increase of over 250% over the unencrypted approach, with RSA having a 36.94% greater difference in cost compared to ECC.

In the results of sending permission for a file, presented in the graphics of Figure 7, it can be observed that the memory usage had a small increase of 1% of the approaches with encryption in relation to the one without encryption, representing approximately 7.5 MB, with the RSA and ECC memory usage being very close.

The execution time had a more expressive increase, being 122.86% for RSA and 103.96% for ECC in relation to the non-encrypted approach, demonstrating a difference between the two of 18.9%. The cost of sending the permission was close to the cost of sending the file, with ECC showing an increase of 280.62%, a difference of 41.62% compared to the cost increase that RSA showed (322.24%).

The difference in cost increase between RSA and ECC in sending data is due to the size of the RSA key, which is relatively larger compared to the ECC implementation in terms of bytes, generating encrypted data with a larger amount of bytes, which ends up impacting the cost that is linked to the amount of information sent to the blockchain.

Analyzing the results obtained with the different files (200 KB, 2 MB, and 30 MB), a difference compatible with the file size was evidenced in the execution time and memory criteria. It is noteworthy that the cost had no increase directly related to the difference in file size, because its variation is due to factors such as: increase in data sent, price of the network “gas” rate, and network occupation.

Even with the memory usage showing little significant difference between the RSA and ECC algorithms, it is possible to observe that ECC presented a more satisfactory execution time and shipping cost increase values, proving to be the algorithm with the best potential to be used within a blockchain-oriented architecture.

## 8. Conclusions and Future Work

This paper presented an architecture that enables the management of the user’s health data storage in a decentralized way through blockchain and using cryptography in order to ensure data privacy, besides placing the control of these data under the user’s responsibility. Regarding cryptography, two approaches were implemented using the RSA and ECC algorithms in order to verify the impact on the architecture. The AES algorithm was used to encrypt the files sent to the IPFS platform.

The development of the architecture by means of the set of technologies and tools described, and with the application of encryption on the sensitive data, can be used and applied within real scenarios, with a low initial implementation cost due to the use of a public blockchain. This paper evaluated the encryption techniques used, seeking to provide a parallel between two of the main asymmetric key algorithms, which can help in the choosing of one of the encryption techniques, depending on the application being built.

Through the results of the tests performed with the two algorithms, it can be observed that cryptography had an impact on the evaluated criteria, mainly in relation to cost, with an increase above 250%, and to execution time, RSA being the algorithm with the greatest increase above 70%. Regarding memory, the difference was less significant between the algorithms, with the increase in memory usage being linked to the size of the file sent. The impact that cryptography had on the architecture is valid and justifiable due to all the privacy and data security that is provided by the combination of architecture and cryptography.

In future works, we consider conducting tests and collecting data to evaluate the performance of other encryption algorithms within the same scenario, and in scenarios using other blockchains as a base. It is also possible to verify the possibility of using anonymization methods, such as k-anonymity and differential privacy, in conjunction with cryptography in the architecture, in order to increase user privacy. Furthermore, as a future work, the safety control of keys in a decentralized way could be added to the application.

## Figures and Tables

**Table 1:** Comparison between the work-related features.

Features	[14]	[15]	[16]	[17]	[33]	[34]	This Work
Blockchain Platform	Hyperledger Fabric	Ethereum	Ethereum	Hyperledger Fabric (Consortium)	Consortium	Ethereum	Ethereum
Healthcare	Yes	Yes	Yes	No	Yes	Yes	Yes
Encryption	No	ECC	Yes (Unknown)	Paillier	CEC-ABE	No	ECC, RSA, AES
Network Type	Permissioned	Permissionless	Permissionless	Permissioned	Permissioned	Permissionless	Permissionless
Anonymity	Anonymiza-tion	Pseudonymity	Pseudonymity	Pseudonymity	Pseudonymity	Pseudonymity	Pseudonymity
DApp	No	Yes	Unknown	Yes	No	Yes	Yes
Off-chain	No	Cloud	Cloud	IPFS	Edge Cloud	IPFS	IPFS

## Data Availability

Not applicable.

## Abbreviations

The following abbreviations are used in this manuscript:

ABI	Application Binary Interface
AES	Advanced Encryption Standard
CID	Content identifier
DApp	Decentralized Application
DDoS	Distributed Denial of Service
ECC	Elliptic Curve Cryptography
ECIES	Elliptic Curve Integrated Encryption Scheme
EVM	Ethereum Virtual Machine
IPFS	InterPlanetary File System
JSON	JavaScript Object Notation
POW	Proof-of-Work
RSA	Rivest-Shamir-Adleman

## Appendix A

In this appendix, the figures for the communication, sending, and receiving of information from IPFS and the Ethereum blockchain can be found.

## Appendix B

In this appendix, the figures related to the RSA cryptography can be found.

## Appendix C

In this appendix, the figures related to the ECC cryptography can be found.

## Appendix D

In this appendix, the figures related to the AES cryptography can be found.
